# Intensive induction chemotherapy with C-BOP/BEP for intermediate- and poor-risk metastatic germ cell tumours (EORTC trial 30948)

**DOI:** 10.1038/sj.bjc.6602830

**Published:** 2005-10-25

**Authors:** S D Fosså, B Paluchowska, A Horwich, G Kaiser, P H M de Mulder, O Koriakine, A T van Oosterom, L de Prijck, L Collette, R de Wit, and the EORTC GU Group

**Affiliations:** 1 Department of Oncology, Norwegian Radium Hospital and University of Oslo, Oslo, Norway; 2 Department of Urology, Maria Sklodowska – Curie Memorial Cancer Center, Warsaw, Poland; 3 Department of Academic Radiotherapy, Royal Marsden Hospital, Sutton, UK; 4 Department of Oncology, Klinikum Nürnberg, Nürnberg, Germany; 5 Department of Internal Medicine, Sint Radboud University Hospital, Nijmegen, The Netherlands; 6 Department of Urology, Medical Radiological Research Center, Obninsk (formerly: Cancer Research Center, Moskow, Russia), Russia; 7 Department of Oncology, Universitair Ziekenhuis Gasthuisberg, Leuven, Belgium; 8 Data Center, Data Management Unit, EORTC Data Center, Brussels, Belgium; 9 Department of Medical Oncology, Erasmus Medical Center, Rotterdam, The Netherlands

**Keywords:** intermediate and poor prognosis metastatic germ cell tumours, bleomycin, carboplatin, vincristine, cisplatin, etoposide

## Abstract

New chemotherapy regimens are continuously explored in patients with high-risk malignant germ cell tumours (MGCTs). This multicentre phase II trial assessed the efficacy and toxicity of C-BOP/BEP chemotherapy in intermediate and poor prognosis MGCT (IGCCCG criteria). C-BOP/BEP treatment consisted of cycles of cisplatin, vincristine, bleomycin and carboplatin, followed by one cycle of vincristine and bleomycin and three cycles of BEP (bleomycon, etoposide, cisplatin). The trial was designed to demonstrate a 1-year progression-free survival rate of 80%, that is, to exclude a 1-year rate of 70% or less, with a one-sided significance level of 5%. Secondary end points included toxicity, overall survival and the postchemotherapy complete response rate. In total, 16 European hospitals entered 66 eligible patients (intermediate prognosis group: 37; poor prognosis group: 29). A total of 45 patients (68.2%, 95% confidence interval (95% CI): 56.9–79.4%) achieved a complete response (intermediate prognosis: 30; poor prognosis: 15). After a median observation time of 40.4 months (range: 13.7–66.3), the 1-year progression-free survival rate was 81.8% 95% CI: 72.5–91.1%). The 2-year overall survival was 84.5% (95% CI: 75.6–93.3%). In all, 51 patients experienced at least one episode of WHO grade 3/4 leucopenia, and at least one event of grade 3/4 thrombocytopenia occurred in 30 patients. There was no toxic death. With an 82% 1-year progression-free survival and a lower limit of the 95% CI above 70%, the efficacy of C-BOP/BEP is comparable to that of published alternative chemotherapy schedules in high-risk MGCT patients. The treatment's toxicity is manageable in a multicentre setting. In poor prognosis patients, C-BOP/BEP should be compared to standard chemotherapy of four cycles of BEP.

In patients with metastatic germ cell tumour (MGCT), four cycles of BEP regimen (bleomycin, etoposide, cisplatin) are considered to be the standard chemotherapy, even in patients belonging to the intermediate and poor prognosis groups defined by the International Germ Cell Collaborative Consensuses Group (ICCCCG) (IGCCCG, 1997). Nevertheless, many attempts have been undertaken to improve the outcome of these patients by the introduction of new chemotherapeutic agents, the use of alternating drug combinations, high-dose chemotherapy schedules or chemotherapy schedules that apply drugs at high density (Table 1). Several research groups have retrospectively categorised their patients by the IGCCCG classification system and have demonstrated survival rates superior to those shown in the first report of the IGCCCG for patients belonging to the poor prognosis group.

Based on the schedule described by Wettlaufer *et al* (1984), the Royal Marsden Hospital in the late 1980s started to develop an ‘intensive induction regimen’ in an effort to overcome rapid tumour cell proliferation (Horwich *et al*, 1989a, 1994b). During the first 4 weeks, cisplatin, vincristine and bleomycin (BOP) were administered with a 7-day interval, followed by a further two cycles of bleomycin and vincristine (BO), and thereafter by three cycles of BEP. The final C-BOP/BEP regimen contained a moderate dose of carboplatin (C) in order to achieve a higher total dose of platinum without increasing toxicity. Of 21 patients treated between 1989 and 1992 at the Royal Marsden, 18 were still alive and disease free after a follow-up of 36 months, resulting in a 2-year survival of 90%. A subsequent extended phase II study confirmed the promising single-centre results in 54 patients with unfavourable risk using the Royal Marsden classification system (Christian *et al* (2003)). This trial performed in three experienced oncological units documented survival rates that were comparable to those achieved by the use of high-dose chemotherapy with autologous haematological stem cell support (Schmoll *et al*, 2003).

Based on these promising results, a prospective multicentre trial using the C-BOP/BEP regimen seemed warranted in patients belonging to the intermediate- and high-risk groups categorised according to the international classification system. Therefore, in 1998, the EORTC GU group decided to further assess the C-BOP/BEP chemotherapy schedule with the aim, in a prospective multicentre setting, to evaluate the regimen's feasibility and toxicity, to estimate the complete response rate and to establish the progression-free and overall survival.

## PATIENTS AND METHODS

### Patients

Eligible patients had histologically confirmed germ cell cancer (seminoma or nonseminoma) and had to be of intermediate or poor prognosis according to the IGCCCG classification system. They should be aged between 18 and 65 years, without major organ dysfunction unless caused by the malignant disease. All patients had to give informed consent, and consent from local ethical boards had to be obtained. Patients with previous chemotherapy and/or a second malignancy, except a basal cell skin cancer, were ineligible as were those with a creatinine clearance below 40 ml/min, unless this was due to obstructive uropathy which could be relieved by nephrostomy or stenting. Previous radiotherapy was not an exclusion criterion.

### Pretreatment evaluation

Pretreatment evaluation comprised physical examination, CT scanning of the abdomen and chest, measurement of serum alpha-foetoprotein (AFP), human choriogonadotropin (HCG) and lactate dehydrogenase (LDH). A CT scan or an MRI scan of the brain had to be performed in patients presenting with CNS symptoms, those with a serum HCG >1 × 10^5^ IU/l or in patients with more than 20 lung metastases.

### Study design

All patients had to be registered at the EORTC Data Center in Brussels before the onset of chemotherapy or latest within the first 2 weeks after start. Chemotherapy comprised an initial intensive induction phase with C-BOP/BO for 6 weeks followed by three 3-weekly cycles of BEP with reduced doses of bleomycin (Table 2).

The C-BOP phase consisted of two identical 2-week cycles. The carboplatin dose applied on day 8 of these 2-week cycles was in milligrams: 3 × (GFR (glomerular filtration rate)+25). Bleomycin 15 mg was applied over 15 min on days 1 and 8. During days 8–12, a total of 75 mg bleomycin was given as continuous infusion (15 mg day^−1^). The two C-BOP cycles were, after 2 weeks, followed by cycle 3, consisting of bleomycin and vincristine. Cycles 4, 5 and 6 of BEP chemotherapy, given with reduced doses of bleomycin (45 mg cycle^−1^), were applied at 3 weekly intervals. Dose reductions during the BEP cycle were planned only if there had been an episode of neutropenic fever or sepsis in the previous cycle, in which case the etoposide was given at 100 mg m^−2^ day^−1^ for only 3 days during the following cycle.

After chemotherapy, all patients with nonseminomatous germ cell tumours and normalised serum tumour markers, but with evidence of residual disease, had to undergo resection of the residual tumour masses. As 80–90% of the postchemotherapy residual masses in patients with seminoma contain completely necrotic tumour tissue and major surgery has been shown to be somehow risky in these cases, routine postchemotherapy surgery was not recommended in seminoma patients.

### Response evaluation

Patients had to be evaluated for response within 4 weeks after commencing cycle 6 or immediately after their postchemotherapy surgery. Patients with normal tumour markers and no clinical or radiological evidence of residual masses were classified as *complete responders.* If ‘no tumour’, ‘necrosis only’ or ‘mature teratoma only’ was detected in the completely resected specimen, the case was also categorised as *complete response*.

Patients who after six cycles had persistently elevated tumour markers, although reduced compared to the pretreatment values, and/or who had residual vital malignant tumour in the operation specimen belonged to the category of *incomplete response. Progression* at any time after start of C-BOP/BEP was defined as rising serum tumour markers above the upper limit of the institution's normal range or occurrence of new metatstatic lesions or by a ⩾25% size increase of pretreatment tumour masses. The rare development of a growing teratoma, documented by histological examination, represented an exception from this definition. Patients who went off study before they had completed the six cycles due to early death due to MGCT or due to progression were categorised as *treatment failures*. Patients with normal serum tumour markers after chemotherapy, but with residual masses, who did not undergo postchemotherapy surgery were regarded as *inevaluable for respons*e but were included in the evaluation of the response rate.

### Follow-up

Completely responding patients and those inevaluable for response did not receive further treatment, whereas those with incomplete response, treatment failure or progression were treated according to the clinical investigator's discretion. Data on type and outcome of such salvage treatment were, however, not collected within the trial.

All patients were to be followed-up for the event of progression. Follow-up examinations were to be performed with 2–3-month intervals during the first year and with increasing intervals thereafter. Biopsy was recommended in seminoma patients with residual masses if no size reduction had recurred after 6 months observation. After progression, all patients were followed for survival.

### Statistical considerations

The primary trial end point was the 1-year progression-free survival rate. The trial was designed to exclude a 1-year rate of 70% or less with 95% confidence interval (95% CI) under the hypothesis that the true rate was 80% (i.e. a lower limit of the 95% CI for the 1-year progression-free survival rate >70%). Under this condition, 62 evaluable patients had to be treated in this study. Using the Kaplan–Meier technique, overall and progression-free survival were calculated from the first day of chemotherapy to the date of death (or progression) or to the date of most recent follow-up, whichever came first.

### Disclosure

This publication was supported by grant numbers 5U10-CA11488-24 to 5U10-CA11488-35 from the National Cancer Institute (Bethesda, Maryland, USA). Its contents are solely the responsibility of the authors, and do not necessarily represent the official views of the National Cancer Institute.

## RESULTS

From June 1996 until March 1998, 16 institutions registered 79 patients; 13 of them were eventually deemed ineligible (registration >14 days after treatment start: 11; erroneous prognosis-group classification: two). Of the 66 eligible patients, 39 belonged to the intermediate prognosis group and 27 to the poor prognosis group (Table 3). The median age of the 65 males and one female (ovarian cancer) subject was 29 years (range 18–50). One patient in the intermediate prognosis group had relapsed with bone metastases after radiotherapy for stage 1 seminoma, and one patient with a poor prognosis nonseminoma had radiotherapy 1 week before trial entry due to imminent spinal cord compression. The median observation time was 40.4 months (range: 13.7–66.3 months). All but six patients were observed to death or a minimum of 2 years.

### Treatment

Of the 66 patients, 62 received all six planned cycles (Table 4). One patient died of progressive MGCT after the first cycle, and three other men progressed after five treatment cycles. The relative median cumulative dose of the four drugs of the C-BOP phase was >90% of the expected dose, as compared to 86% for each of the three drugs of the BEP schedule. Dose modifications were frequently required (postponement of at least one cycle: 33 patients; dose reduction during at least one cycle: seven patients; discontinuation of a drug during at least one cycle: seven patients; premature discontinuation of at least one cycle: 13 patients).

Overall, haematological toxicity was the most frequent reason for dose modification (30 patients). Pulmonary and renal toxicity led to dose modification in five and four patients, respectively. Resections of postchemotherapy residual masses were performed in 44 patients (intermediate-risk group: 25; high-risk group: 19). Viable cancer cells were found in one patient from each risk group. Of the 44 patients, 19 displayed postchemotherapy necrosis/fibrosis, and 22 had mature teratoma (50%), without statistical difference between the two subgroups as to histopathological outcome.

### Outcome

Immediately after chemotherapy or after their postchemotherapy surgery, 45 of all 66 patients (68.2%, 95% CI: 56.9–79.4%) had a complete response (intermediate group: 30 patients (76%); high-risk group: 15 patients (56%)). Treatment failure was recorded in the four patients who did not complete the six planned cycles. Incomplete response was recorded in six patients, and 11 were inevaluable for response. At the end of the observation time, progression had occurred in a total of 17 patients (26%); intermediate-risk group: five patients; high-risk group: 12 patients, including the four patients with treatment failure. At the date of last observation, 55 patients were alive and 11 were dead, 10 of them due to their MGCT. (The 11th patient died tumour-free in a car accident.)

**Figure 1:**
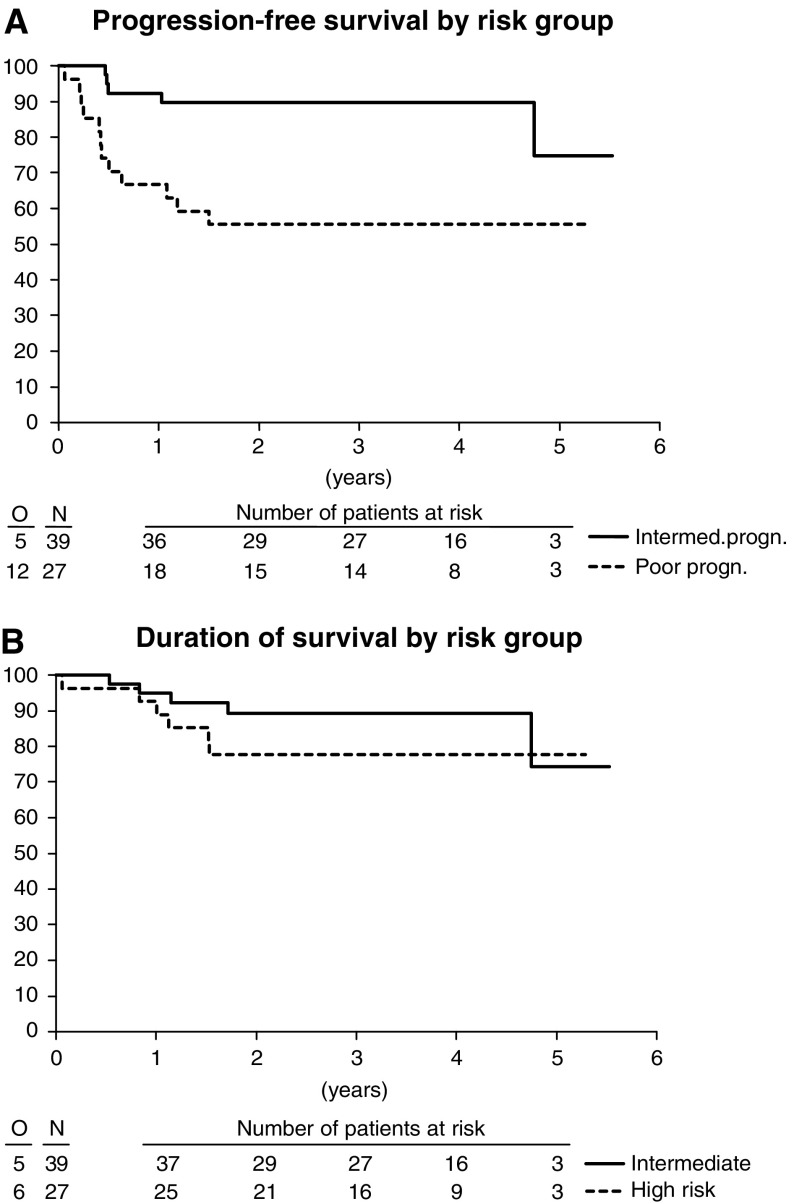
Progression-free (**A**) and overall (**B**) survival in patients with MGCT of the intermediate (37 patients) and poor prognosis (29 patients) group.

The 1-year progression-free survival for all patients was 81.8% (95% CI: 72.5–91.1%). The 2-year progression-free survival for the intermediate- and high-risk group was 89.7% (95% CI: 80.2–99.3%) and 55.6% (95% CI: 36.8–74.3%), respectively (Figure 1A). The 1- and 2-year overall survivals were, respectively, 93.9% (95% CI: 88.2–99.7%) and 84.5% (95% CI: 75.6–93.3%) (Figure 1B).

### Toxicity

A total of 59 patients experienced grade 3 or 4 toxicity at least once (Table 4). Grade 4 haematological toxicity was seen in 40.9% of the patients, with thrombocytopenia being the most frequent side effect. Four patients developed grade 3/4 pulmonary toxicity and 14 patients developed at least one period of grade 3/4 infection or febrile neutropenia. There was no toxic death. G-CSF support was administered at least once to 23 patients.

## DISCUSSION

With an 81.8% observed 1-year progression-free survival and a lower limit of the 95% CI above 70%, this phase II study reached its target demonstrating results of a true 1-year progression-free survival rate of >80%. The 2-year overall survival rates in, respectively, intermediate and poor prognosis patients were 89 and 78%. The C-BOP/BEP schedule was feasible in a multicentre setting with predictable and manageable short-term toxicity without any toxic death.

In patients with metastatic germ cell tumours of unfavourable prognosis, attempts to improve the outcome of the standard regimen BEP as induction chemotherapy have focused on three approaches: firstly, intensification of cisplatin application; secondly, introduction of new drugs together with the sequential use of alternating drug combinations; and thirdly, increasing the cumulative doses of the cytotoxic, drugs including the use of high-dose chemotherapy followed by autologous stem cell support. Not rarely, clinical investigators have combined these strategies.

Intensification did not improve the 1-year failure-free survival rate in the EORTC GU group's phase III study that in poor prognosis patients (Royal Marsden Hospital categorisation system) compared three cycles of BOP (bleomycin, vincristine, cisplatin) given every 10 days followed by three cycles of VIP-B (etoposide, ifosfamide, cisplatin, bleomycin) with four cycles BEP plus two cycles of EP (Kaye *et al*, 1998). Concerning the introduction of new agents, four courses of VIP were compared with four cycles of BEP (de Wit *et al*, 1998b), but both regimens led to similar 2-year failure-free survival (VIP: 64%; BEP: 60%) and 2-year overall survival (VIP: 74%; BEP: 71%). The greatest experience with the use of alternative drugs has been with the POMB/ACE regimen (cisplatin, vincristine, methotrexate,bleomycin, actinomycin-D, cyclophosphamide, etoposide) with two institutions treating 339 patients with MGCT over a 20-year period with a median follow-up of 8 years (Bower *et al*, 1997). In total, 92 patients were retrospectively identified as IGCCC poor prognosis. The POMB-EPI regimen, essentially consisting of the components of POMB alternating with a modified VIP regimen, resulted in a 2-year overall survival of 64% in 22 patients within the poor prognosis group (Germa-Lluch *et al*, 1999). Taxanes in combination with cisplatin-based chemotherapy have also been tested. In a phase I/II study with BEP combined with Taxol, all 13 evaluable patients with intermediate or poor prognosis MGCT achieved a complete response and none of these patients relapsed with a median follow-up of 18 months (de Wit *et al*, 1999a). Currently the EORTC GU group conducts a phase III study comparing four cycles of T-BEP with four cycles of BEP in patients with intermediate prognosis features. Fizazi *et al* (2002a) reported a 5-year overall survival of 88% in the intermediate prognosis group and 83% (95% CI: 58–100%) in the poor prognosis group using the CISCA/VB regimen (cisplatin, cyclophosphamide, adriamycin, vinblastine, bleomycin). The same group described a phase II study using the BOP-CISCA-POMB-ACE regimen comprising eight drugs plus granulocyte colony-stimulating factor (Fizazi *et al*, 2002b). Investigators of the Genitourinary Group of the French Federation of Cancer Centers have embarked on a prospective trial of BEP *vs* CISCA (II)/VB (IV) in poor-risk patients (Culine *et al*, 1997). The third approach implies the attempts to use high-dose combination chemotherapy with autologous haematopoetic stem cell support. In several phase II trials, this strategy has resulted in promising survival rates with acceptable toxicity without toxic death, for example, given as CEC (cisplatin, etoposide, cyclophosphamide) after induction with BEP (Decatris *et al*, 2000), or as sequential high-dose VIP plus paclitaxel (Hartmann *et al*, 2002). The German Testicular Cancer Group reported a 79% 2-year survival in 182 poor prognosis patients (IGCCCG criteria) who were treated with escalating doses of VIP followed by autologous stem cell support (Schmoll *et al*, 2003). In poor prognosis patients, high-dose schedules using CEC or VIP together with autologous blood stem cell transplantation are currently investigated in prospective randomised trials by the US Intergroup (BEP *vs* high-dose CEC) and the EORTC GU Group (BEP *vs* high-dose VIP).

The C-BOP/BEP schedule used in this study principally comprises two manoeuvers to improve the efficacy of induction chemotherapy during the first 6 weeks: an increase of the cumulative cisplatin dose and frequent cycling of the cisplatin, vincristine and bleomycin in an effort to overcome rapid proliferation, together with alternating the C-BOP schedule with BEP. For example, in the C-BOP schedule, the dose intensity of platinum during the first 6 weeks is 63 mg m^−2^ week^−1^ (based on a carboplatin AUC3 being equivalent to cisplatin 50 mg m^−2^) compared to 50 mg m^−2^ week^−1^ in the BOP/VIP schedule and 33.3 mg m^−2^ week^−1^ in the standard BEP. The results of the present study are promising. When C-BOP/BEP is given in a multicentre setting, the complete response rates and the 2-year overall survival rates for, respectively, patients with intermediate and poor prognosis MGCT is at least comparable with the results obtained with other intensive cytotoxic regimens (Table 1). Our 2-year overall survival in the poor prognosis group may seem slightly inferior to Christian *et al*'s figure of 88% achieved at three large cancer centres. This may be due to differences between poor prognosis patients with MGCT as to the type and number of poor risk factors (Decatris *et al*, 2000). Interstudy differences in outcome may reflect differences in the prognostic features or patient selection rather than real differences in treatment efficacy. Christian *et al*'s slightly superior survival figure can also be viewed as a consequence of broader experience with MGCT patients in general at each of the three involved cancer centres as compared to the experience of institutions with few cases of MGCT (Aass *et al*, 1991; Collette *et al*, 1999).

The C-BOP/BEP schedule has been shown to be safe in a multicentre design, with no toxic deaths in the current study. Based on nonrandomised historical studies (International Germ Cell Cancer Collaborative Group, 1997), its superior efficacy compared to BEP cannot be excluded. During the first weeks of the induction chemotherapy, the proportion of myelotoxic drugs in C-BOP is less than in the BEP schedule. This may be advantageous in patients who are very sick due to a large tumour burden at the time of diagnosis and who are at particularly high risk of neutropenic fever and sepsis. Nevertheless, the occurrence of ‘any grade 3/4 toxicity’ in 90% of the patients and of grade 3/4 haematological toxicity in 77% warrants that these patients should be managed at institutions with experience in the treatment of MGCT.

Until the ongoing three phase III studies comparing four cycles of BEP with experimental chemotherapy regimens are finalised, BEP remains the standard treatment of MGCT, even in patients with intermediate and poor prognosis features. The current and published results with c-BOP/BEP support ongoing plans to conduct a multicentre phase III study comparing C-BOP with four cycles of BEP in poor prognosis patients.

### Contributions

The following investigators and centres contributed patients to the study:

SD Fossa, Norwegian Radium Hospital, Oslo, Norway (21); B Paluchowska, Maria Sklodowska – Curie Memorial Cancer Center, Warsaw, Poland (16); A Horwich, Royal Marsden Hospital, Sutton, UK (13); G Kaiser, Klinikum Nürnberg, Nürnberg, Germany (8); P de Mulder, Sint Radboud University Hospital, Nijmegen, The Netherlands (4); O Koriakine, Medical Radiological Research Center, Obninsk, Russia (formerly: Cancer Research Center, Moskow, Russia) (3); ATM Van Oosterom, Universitair Ziekenhuis Gasthuisberg, Leuven, Belgium (3); C Sternberg, San Camillo and Forlanini Hospitals (formerly: San Raffaele Hospital), Roma, Italy (2); G Mead, Royal South Hants Hospitaln Southampton, UK (2); JB Vermorken, Universitair Ziekenhuis Antwerpen, Edegem, Belgium (1); JJ Croles, Bosh Medicentrum, s'Hertogenbosch, The Netherlands (1); R de Wit, Erasmus Medical Center, Rotterdam, The Netherlands (1); HJ Keizer, University Hospital, Leiden, The Netherlands (1); A Bono, Ospedale di Circolo e Fundacione Macchi, Varese, Italy (1); M Williams, Addenbrooke National Health Service, Cambridge, UK (1); RE Coleman, Weston Park Hospital, Sheffield, UK (1).

L de Prijck was the data manager at the EORTC Data Centre, responsible for this trial. L Collette was the statistician at the Data Centre.

## Figures and Tables

**Table 1:** Chemotherapy and outcome in patients with metastatic germ cell tumours belonging to the intermediate- and high-risk group (IGCCCG criteria)

						**Progr.-free survival**		**Overall survival**	
**Year**	**Author**	**Treatment**	**No. of Institutions**	**No. of Patients**	**CR rate (%)**	**Interval (years)**	**Per cent (%)**	**Interval (years)**	**Per cent (%)**
*Intermediate prognostic group*									
1997	IGCCCG	Multiple mostly ‘conventional’	∼30	1524	NA	5	75	5	79
1997	Bower	POMB/ACE	2	41	NA	NA	NA	3	88
2001	Sonnenveld	Multiple	1	105	NA	NA	NA	10	1977–1986: 74
									1987–1996: 87
2002a	Fizazi	CISCA/VB	1	38	NA	NA	NA	5	88
2002b	Fizazi	BOP-CISCA	1	19	NA	3	83	3	83
		POMB-ACE							
2003	Hinton	BEP or VIP	5	84	NA	5	BEP: 84	5	BEP: 84
							VIP: 72		VIP: 77
2004	Anthoney	BOP/BEP	4	27	52	3	79	3	75
2005	Current	C-BOP/BOP	16	37	76	2	90	2	89

*Poor prognostic group*									
1997	IGCCCG	Multiple mostly ‘conventional’	∼30	821	NA	5	41	5	48
1997	Bower	POMB/ACE	2	92	NA	NA	NA	3	75
1999	Germa/Lluch	POMB-Epi	11	22	49	2	58	2	64
1999	DeWit	Taxol/BEP	13	2	100	1.5	100	1.5	100
2000	Decatris	BEP-CEC	1	20	NA	NA	NA	4	66
2001	Sonnenveld	Multiple	1	49	NA	NA	NA	10	1977–1986: 30
									⩾1986–1996: 62
									Total: 42
2002a	Fizazi	CISCA/VB	1	NA	NA	NA	NA	5	83
2002b	Fizazi	BOP-CISCA	1	38	NA	3	65	3	67
		POMB-ACE							
2003	Hinton	BEP or VIP	5	57	NA	5	BEP: 49	5	BEP: 57
							VIP: 56		VIP: 62
2003	Schmoll	VIP-high dose	25	182	66	2	69	2	79
					(85)				
2003	Huddart	CBOP-BEP	3	54	30	3	83	3	88
2004	Anthoney	BOP-BEP	4	19	NA	3	84	5	65
2004	Rosti	Carbo-PEC+Others+high dose	6	22	76	2	67	2	81
2005	Current	CBOP-BEP	16	29	56	2	56	2	78

**Table 2:** C-BOP/BEP treatment regimen

*Cycles 1 and 2: C-BOP (2 weeks each)*
Cisplatin 50 mg/qm days 1 and 2
Vincristine 2 mg i.v. days 1 and 8
Bleomycin 15 mg days 1 and 8
Cisplatin 40 mg/qm+carboplatin AUC × 3 day 8
Bleomycin 15 mg continuous infusion days 8–12

*Cycle 3 BO (2 weeks)*
Vincristine 2 mg i.v., days 1 and 8
Bleomycin 15 mg, days 1 and 8

*Cycle 4,5,6: modified BEP (3 weeks each)*
Cisplatin 20 mg/m^2^/day, days 1–5
Etoposide 100 mg/m^2^/day, days 1–5
Bleomycin 15 mg, days 1, 8, 15

Weeks 1 - - - - - 4 5 6 7 10 13 17
C-BOP BO BO BEP BEP BEP±surgery/FU

**Table 3:** Patient characteristics

	**Risk group**		
	**Intermediate**	**High**	**Total**
*Number of patients entered*	48	31	79^b^
Ineligible^a^	9	4	13
Eligible (years)	39	27	66

*Age (median, range)*	29 (18–50)	28 (18–41)	29 (18–50)
Site			
Testis	35	17	52
Extragonadal	4	10	14
Retroperitoneal	4	4	8
Mediastinal	0	4	4
Other^b^	0	2	2

*Histology*			
Seminoma	2	0	2
Nonseminoma	37	27	64

Previous radiotherapy	1	1	2
*Tumour sites at start of treatment*			
Abdominal LN^c^	37	22	59
Mediastinal LN	5	11	16
Supraclav LN	4	3	7
Lung	16	13	29
Liver	0	9	9
Bone	1	0	1
Brain	0	2	2
Other	2	1	3

a Owing to registration >2 weeks after treatment start (11 patients) or inappropriate serum marker values (2).

b Ovary (1) and abdominal (1).

c Lymph nodes.

**Table 4:** Maximal acute toxicity

	**Number of patients (%)**			
	**WHO 0–1**	**2**	**3**	**4**
Haemoglobin	6	25	27 (40.9)	8 (12.1)
Neutrophils^a^	43	3	9 (13.6)	9 (13.6)
Platelets	23	13	12 (18.2)	18 (27.3)
WBC	4	11	40 (60.6)	11 (16.7)

Infection	44	15	4 (6.1)	3 (4.5)
Febrile neutropenia	51	5	8 (12.1)	2 (3.0)

Mucositis	49	12	3 (4.5)	2 (3.0)
Ototoxicity^b^	56	9		
Sensory neuropathy	52	10	3 (4.5)	1 (1.5)
Pulmonary toxicity	57	5	2 (3.0)	2 (3.0)
Cutaneous toxicity	52	12	1 (1.5)	1 (1.5)

Any haematological toxicity	0	9	27 (4.9)	30 (45.5)

Any nonhaematological toxicity^c^	8	25	25 (37.9)	8 (12.1)
Any toxicity^c^	0	7	27 (40.9)	32 (48.5)

a Missing in two patients.

b Missing in one patient.

c Also including fatigue, nausea, vomiting, diarrhoea, constipation, other neurological toxicity and other side effects.
